# (*E*)-2-Furyl methyl ketone 2,4-dinitro­phenyl­hydrazone

**DOI:** 10.1107/S1600536808015122

**Published:** 2008-05-24

**Authors:** Shang Shan, Yu-Liang Tian, Shan-Heng Wang, Wen-Long Wang, Ying-Li Xu

**Affiliations:** a College of Chemical Engineering and Materials Science, Zhejiang University of Technology, People’s Republic of China

## Abstract

Crystals of the title compound, C_12_H_10_N_4_O_5_, were obtained from a condensation reaction of 2,4-dinitro­phenyl­hydrazine and 2-furyl methyl ketone. The mol­ecule displays a nearly planar structure, and the furan ring is slightly twisted by a dihedral angle of 12.62 (6)° with respect to the phenyl­hydrazone plane. The face-to-face separation of 3.287 (7) Å between parallel benzene rings of adjacent mol­ecules indicates the existence of π–π stacking between dinitro­phenyl rings in the crystal structure.

## Related literature

For general background, see: Okabe *et al.* (1993 ▶); Shan *et al.* (2003*a* ▶, 2006 ▶). For related structures, see: Vickery *et al.* (1985 ▶); Fan *et al.* (2004 ▶); Shan *et al.* (2003*b* ▶).
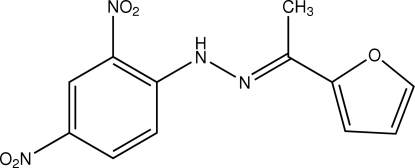


## Experimental

### 

#### Crystal data

C_12_H_10_N_4_O_5_
*M* *_r_* = 290.24
Monoclinic, 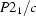

*a* = 9.8917 (8) Å
*b* = 12.8477 (15) Å
*c* = 10.6549 (12) Å
β = 111.63 (2)°
*V* = 1258.7 (3) Å^3^
*Z* = 4
Mo *K*α radiation
μ = 0.12 mm^−1^
*T* = 293 (2) K
0.36 × 0.23 × 0.18 mm

#### Data collection

Rigaku R-AXIS RAPID IP diffractometer
Absorption correction: none
12121 measured reflections
2858 independent reflections
1784 reflections with *I* > 2σ(*I*)
*R*_int_ = 0.026

#### Refinement

*R*[*F* ^2^ > 2σ(*F* ^2^)] = 0.038
*wR*(*F* ^2^) = 0.111
*S* = 1.03
2858 reflections
192 parameters
H-atom parameters constrained
Δρ_max_ = 0.21 e Å^−3^
Δρ_min_ = −0.17 e Å^−3^

### 

Data collection: *PROCESS-AUTO* (Rigaku, 1998 ▶); cell refinement: *PROCESS-AUTO*; data reduction: *CrystalStructure* (Rigaku/MSC, 2002 ▶); program(s) used to solve structure: *SIR92* (Altomare *et al.*, 1993 ▶); program(s) used to refine structure: *SHELXL97* (Sheldrick, 2008 ▶); molecular graphics: *ORTEP-3 for Windows* (Farrugia, 1997 ▶); software used to prepare material for publication: *WinGX* (Farrugia, 1999 ▶).

## Supplementary Material

Crystal structure: contains datablocks I, global. DOI: 10.1107/S1600536808015122/om2233sup1.cif

Structure factors: contains datablocks I. DOI: 10.1107/S1600536808015122/om2233Isup2.hkl

Additional supplementary materials:  crystallographic information; 3D view; checkCIF report

## Figures and Tables

**Table 1:** Hydrogen-bond geometry (Å, °)

*D*—H⋯*A*	*D*—H	H⋯*A*	*D*⋯*A*	*D*—H⋯*A*
N3—H3⋯O1	0.86	1.97	2.6063 (15)	130
C9—H9⋯O4^i^	0.93	2.41	3.334 (2)	172
C11—H11⋯O2^ii^	0.93	2.41	3.1530 (19)	137

Symmetry codes: (i) 
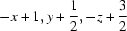
; (ii) 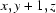.

**Fig. 1.**
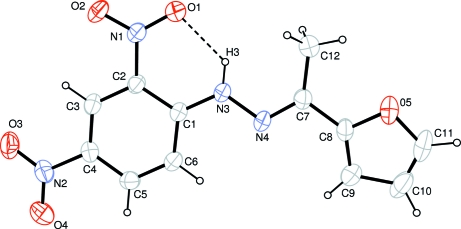
The molecular structure of the title compound with 30% probability displacement ellipsoids for non-H atoms.

**Fig. 2.**
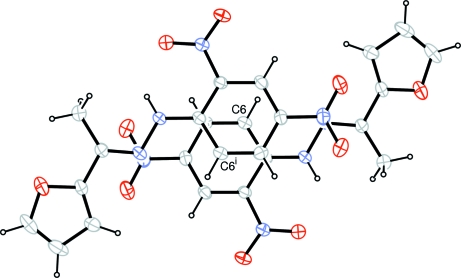
A diagram showing the overlapped arrangement of parallel benzene rings of neighboring molecules [symmetry code: (i) 1 - x,1 - y,1 - z].

## supplementary crystallographic information

### Comment

As some phenylhydrazone derivatives have shown to be potentially DNA damaging and mutagenic agents (Okabe *et al.*, 1993), a series of new phenylhydrazone derivatives have been synthesized in our laboratory (Shan *et al.*, 2003*a*; Shan *et al.*, 2006). As part of the ongoing investigation, the title compound has recently been prepared and its crystal structure is reported here.

The molecular structure of the title compound is shown in Fig. 1. The molecule displays an approximately planar structure, the furan ring is slightly twisted with respect to the phenylhydrazone plane with a dihedral angle of 12.62 (6)°. The N4—C7 bond distance (Table 1) indicates a typical C=N double dond. The molecule assumes an E configuration with the phenylhydrazine and furan located on the opposite sides of the C=N bond. An intramolecular hydrogen bond is observed between the N3-imino and the adjacent N1-nitro groups; such a hydrogen bonding is a common feature in *o*-nitrophenylhydrazine compounds (Vickery *et al.*, 1985; Fan *et al.*, 2004).

A partially overlapped arrangement of parallel benzene rings of adjacent molecules is illustrated in Fig. 2. The face-to-face separation of 3.287 (7) Å strongly indicates the existence of π-π stacking between parallel dinitrophenyl rings of adjacent molecules in the crystal. It agrees with that found in a related dinitrophenylhydrazine compound, isobutylaldehyde 2,4-dinitrophenylhydrazone (Shan *et al.*, 2003*b*).

The crystal structure also contains intermolecular weak C—H···O hydrogen bonding (Table 2).

### Experimental

2,4-Dinitrophenylhydrazine (0.4 g, 2 mmol) was dissolved in ethanol (10 ml), and H_2_SO_4_ solution (98%, 0.5 ml) was slowly added to the ethanol solution with stirring. The solution was heated at 333 K for several min until the solution cleared. 2-Furyl methylketone (0.22 g, 2 mmol) was added to the above solution with continuous stirring, and the mixture was refluxed for 30 min. When the solution had cooled to room temperature brown powder crystals appeared. The powder crystals were separated and washed with water three times. Recrystallization from an absolute ethanol solution yielded well shaped single crystals.

### Refinement

Methyl H atoms were placed in calculated positions with C—H = 0.96 Å and the torsion angle was refined to fit the electron density, *U*_iso_(H) = 1.5*U*_eq_(C). Other H atoms were placed in calculated positions with C—H = 0.93 and N—H = 0.86 Å, and refined in riding mode, *U*_iso_(H) = 1.2*U*_eq_(C,*N*).

### Crystal data

**Table:**

C_12_H_10_N_4_O_5_	*F*_000_ = 600
*M**_r_* = 290.24	*D*_x_ = 1.532 Mg m^−^^3^
Monoclinic, *P*2_1_/*c*	Mo *K*α radiation λ = 0.71073 Å
Hall symbol: -P 2ybc	Cell parameters from 5266 reflections
*a* = 9.8917 (8) Å	θ = 3.2–26.0º
*b* = 12.8477 (15) Å	µ = 0.12 mm^−^^1^
*c* = 10.6549 (12) Å	*T* = 293 (2) K
β = 111.63 (2)º	Prism, brown
*V* = 1258.7 (3) Å^3^	0.36 × 0.23 × 0.18 mm
*Z* = 4	

### Data collection

**Table:**

Rigaku R-AXIS RAPID IP diffractometer	2858 independent reflections
Radiation source: fine-focus sealed tube	1784 reflections with *I* > 2σ(*I*)
Monochromator: graphite	*R*_int_ = 0.026
Detector resolution: 10.00 pixels mm^-1^	θ_max_ = 27.4º
*T* = 293(2) K	θ_min_ = 3.2º
ω scans	*h* = −12→12
Absorption correction: none	*k* = −16→16
12121 measured reflections	*l* = −13→13

### Refinement

**Table:**

Refinement on *F*^2^	Hydrogen site location: inferred from neighbouring sites
Least-squares matrix: full	H-atom parameters constrained
*R*[*F*^2^ > 2σ(*F*^2^)] = 0.038	*w* = 1/[σ^2^(*F*_o_^2^) + (0.0649*P*)^2^] where *P* = (*F*_o_^2^ + 2*F*_c_^2^)/3
*wR*(*F*^2^) = 0.111	(Δ/σ)_max_ < 0.001
*S* = 1.03	Δρ_max_ = 0.21 e Å^−^^3^
2858 reflections	Δρ_min_ = −0.17 e Å^−^^3^
192 parameters	Extinction correction: SHELXL97 (Sheldrick, 2008), Fc^*^=kFc[1+0.001xFc^2^λ^3^/sin(2θ)]^-1/4^
Primary atom site location: structure-invariant direct methods	Extinction coefficient: 0.0047 (11)
Secondary atom site location: difference Fourier map	

### Special details

**Table:**

Geometry. All e.s.d.'s (except the e.s.d. in the dihedral angle between two l.s. planes) are estimated using the full covariance matrix. The cell e.s.d.'s are taken into account individually in the estimation of e.s.d.'s in distances, angles and torsion angles; correlations between e.s.d.'s in cell parameters are only used when they are defined by crystal symmetry. An approximate (isotropic) treatment of cell e.s.d.'s is used for estimating e.s.d.'s involving l.s. planes.
Refinement. Refinement of *F*^2^ against ALL reflections. The weighted *R*-factor *wR* and goodness of fit *S* are based on *F*^2^, conventional *R*-factors *R* are based on *F*, with *F* set to zero for negative *F*^2^. The threshold expression of *F*^2^ > σ(*F*^2^) is used only for calculating *R*-factors(gt) *etc*. and is not relevant to the choice of reflections for refinement. *R*-factors based on *F*^2^ are statistically about twice as large as those based on *F*, and *R*- factors based on ALL data will be even larger.

### Fractional atomic coordinates and isotropic or equivalent isotropic displacement parameters (Å^2^)

**Table:**

	*x*	*y*	*z*	*U*_iso_*/*U*_eq_	
O1	0.13582 (13)	0.43547 (8)	0.14104 (11)	0.0648 (4)	
O2	0.15573 (13)	0.27996 (8)	0.22020 (12)	0.0648 (4)	
O3	0.44159 (13)	0.19704 (8)	0.66737 (13)	0.0660 (4)	
O4	0.52296 (15)	0.31707 (10)	0.81753 (13)	0.0742 (4)	
O5	0.09817 (11)	0.93990 (8)	0.18361 (12)	0.0566 (3)	
N1	0.17859 (13)	0.37357 (9)	0.23615 (12)	0.0452 (3)	
N2	0.45693 (14)	0.28930 (10)	0.70150 (14)	0.0498 (3)	
N3	0.21841 (13)	0.59367 (8)	0.30483 (12)	0.0412 (3)	
H3	0.1757	0.5753	0.2219	0.049*	
N4	0.22992 (13)	0.69747 (8)	0.33900 (12)	0.0411 (3)	
C1	0.27421 (13)	0.52042 (9)	0.40146 (14)	0.0349 (3)	
C2	0.25858 (14)	0.41229 (10)	0.37100 (14)	0.0368 (3)	
C3	0.31897 (14)	0.33809 (10)	0.46968 (15)	0.0406 (3)	
H3A	0.3085	0.2677	0.4478	0.049*	
C4	0.39417 (14)	0.36870 (10)	0.59975 (15)	0.0403 (3)	
C5	0.40955 (15)	0.47424 (11)	0.63469 (15)	0.0412 (3)	
H5	0.4597	0.4940	0.7238	0.049*	
C6	0.35072 (14)	0.54818 (10)	0.53751 (14)	0.0393 (3)	
H6	0.3613	0.6182	0.5615	0.047*	
C7	0.16518 (14)	0.76093 (10)	0.24031 (14)	0.0395 (3)	
C8	0.18273 (16)	0.87045 (10)	0.27796 (16)	0.0431 (4)	
C9	0.26625 (19)	0.92158 (12)	0.38905 (18)	0.0586 (4)	
H9	0.3329	0.8926	0.4674	0.070*	
C10	0.2326 (2)	1.02927 (13)	0.3632 (2)	0.0691 (5)	
H10	0.2734	1.0842	0.4218	0.083*	
C11	0.1335 (2)	1.03623 (12)	0.2414 (2)	0.0662 (5)	
H11	0.0926	1.0983	0.1997	0.079*	
C12	0.07984 (19)	0.73130 (12)	0.09826 (17)	0.0556 (4)	
H12A	0.1447	0.7073	0.0560	0.083*	
H12B	0.0268	0.7907	0.0502	0.083*	
H12C	0.0130	0.6767	0.0968	0.083*	

### Atomic displacement parameters (Å^2^)

**Table:**

	*U*^11^	*U*^22^	*U*^33^	*U*^12^	*U*^13^	*U*^23^
O1	0.0920 (9)	0.0420 (6)	0.0438 (7)	−0.0007 (6)	0.0055 (6)	−0.0006 (5)
O2	0.0836 (8)	0.0323 (6)	0.0615 (8)	−0.0105 (5)	0.0068 (6)	−0.0098 (5)
O3	0.0778 (8)	0.0408 (6)	0.0728 (9)	0.0058 (5)	0.0200 (7)	0.0147 (5)
O4	0.0884 (9)	0.0708 (8)	0.0459 (8)	0.0053 (7)	0.0041 (7)	0.0107 (6)
O5	0.0572 (6)	0.0371 (6)	0.0754 (8)	0.0069 (5)	0.0242 (6)	0.0136 (5)
N1	0.0513 (7)	0.0333 (6)	0.0443 (8)	−0.0020 (5)	0.0096 (6)	−0.0035 (5)
N2	0.0510 (7)	0.0468 (8)	0.0489 (9)	0.0030 (6)	0.0151 (6)	0.0111 (6)
N3	0.0510 (7)	0.0272 (5)	0.0399 (7)	−0.0003 (5)	0.0103 (5)	−0.0014 (5)
N4	0.0490 (7)	0.0263 (6)	0.0468 (7)	−0.0013 (5)	0.0164 (6)	−0.0013 (5)
C1	0.0349 (7)	0.0296 (6)	0.0404 (8)	−0.0010 (5)	0.0142 (6)	−0.0009 (5)
C2	0.0373 (7)	0.0305 (6)	0.0400 (8)	−0.0020 (5)	0.0112 (6)	−0.0026 (5)
C3	0.0411 (7)	0.0297 (6)	0.0492 (9)	−0.0020 (5)	0.0147 (7)	0.0013 (6)
C4	0.0395 (7)	0.0372 (7)	0.0434 (9)	0.0017 (6)	0.0146 (6)	0.0064 (6)
C5	0.0421 (7)	0.0439 (8)	0.0366 (8)	−0.0028 (6)	0.0132 (6)	−0.0034 (6)
C6	0.0433 (7)	0.0316 (7)	0.0448 (9)	−0.0023 (6)	0.0181 (6)	−0.0047 (6)
C7	0.0428 (7)	0.0343 (7)	0.0427 (8)	−0.0003 (6)	0.0171 (6)	0.0024 (6)
C8	0.0507 (8)	0.0319 (7)	0.0496 (9)	0.0045 (6)	0.0220 (7)	0.0084 (6)
C9	0.0800 (11)	0.0390 (8)	0.0549 (10)	−0.0056 (8)	0.0227 (9)	−0.0049 (7)
C10	0.0959 (14)	0.0354 (8)	0.0892 (15)	−0.0106 (8)	0.0495 (13)	−0.0130 (9)
C11	0.0762 (12)	0.0289 (8)	0.1072 (18)	0.0051 (8)	0.0499 (13)	0.0085 (9)
C12	0.0682 (10)	0.0446 (8)	0.0471 (10)	0.0010 (8)	0.0133 (8)	0.0036 (7)

### Geometric parameters (Å, °)

**Table:**

O1—N1	1.2338 (16)	C3—H3A	0.9300
O2—N1	1.2239 (15)	C4—C5	1.3995 (19)
O3—N2	1.2327 (17)	C5—C6	1.367 (2)
O4—N2	1.2194 (18)	C5—H5	0.9300
O5—C11	1.369 (2)	C6—H6	0.9300
O5—C8	1.3735 (18)	C7—C8	1.4560 (19)
N1—C2	1.4491 (18)	C7—C12	1.485 (2)
N2—C4	1.4510 (18)	C8—C9	1.339 (2)
N3—C1	1.3533 (17)	C9—C10	1.426 (2)
N3—N4	1.3760 (15)	C9—H9	0.9300
N3—H3	0.8600	C10—C11	1.309 (3)
N4—C7	1.2977 (18)	C10—H10	0.9300
C1—C6	1.4115 (19)	C11—H11	0.9300
C1—C2	1.4221 (17)	C12—H12A	0.9600
C2—C3	1.3813 (19)	C12—H12B	0.9600
C3—C4	1.367 (2)	C12—H12C	0.9600
			
C11—O5—C8	105.81 (13)	C4—C5—H5	120.1
O2—N1—O1	121.97 (13)	C5—C6—C1	121.31 (13)
O2—N1—C2	118.71 (12)	C5—C6—H6	119.3
O1—N1—C2	119.32 (11)	C1—C6—H6	119.3
O4—N2—O3	122.83 (14)	N4—C7—C8	114.26 (13)
O4—N2—C4	118.26 (13)	N4—C7—C12	126.16 (13)
O3—N2—C4	118.91 (14)	C8—C7—C12	119.58 (12)
C1—N3—N4	120.13 (12)	C9—C8—O5	109.82 (13)
C1—N3—H3	119.9	C9—C8—C7	133.59 (14)
N4—N3—H3	119.9	O5—C8—C7	116.59 (13)
C7—N4—N3	115.25 (12)	C8—C9—C10	106.32 (17)
N3—C1—C6	121.30 (12)	C8—C9—H9	126.8
N3—C1—C2	121.75 (13)	C10—C9—H9	126.8
C6—C1—C2	116.95 (12)	C11—C10—C9	107.09 (16)
C3—C2—C1	121.36 (13)	C11—C10—H10	126.5
C3—C2—N1	116.28 (12)	C9—C10—H10	126.5
C1—C2—N1	122.37 (12)	C10—C11—O5	110.96 (14)
C4—C3—C2	119.62 (13)	C10—C11—H11	124.5
C4—C3—H3A	120.2	O5—C11—H11	124.5
C2—C3—H3A	120.2	C7—C12—H12A	109.5
C3—C4—C5	120.90 (13)	C7—C12—H12B	109.5
C3—C4—N2	118.57 (13)	H12A—C12—H12B	109.5
C5—C4—N2	120.52 (13)	C7—C12—H12C	109.5
C6—C5—C4	119.84 (13)	H12A—C12—H12C	109.5
C6—C5—H5	120.1	H12B—C12—H12C	109.5

### Hydrogen-bond geometry (Å, °)

**Table:**

*D*—H···*A*	*D*—H	H···*A*	*D*···*A*	*D*—H···*A*
N3—H3···O1	0.86	1.97	2.6063 (15)	130
C9—H9···O4^i^	0.93	2.41	3.334 (2)	172
C11—H11···O2^ii^	0.93	2.41	3.1530 (19)	137

Symmetry codes: (i) −*x*+1, *y*+1/2, −*z*+3/2; (ii) *x*, *y*+1, *z*.
